# Aqua­(2-oxido-2,2-diphenyl­acetato-κ^2^ *O* ^1^,*O* ^2^)(1,10-phenanthroline-κ^2^ *N*,*N*′)copper(II)

**DOI:** 10.1107/S1600536809053483

**Published:** 2009-12-16

**Authors:** Xiao-Xia Yang, Fu-Yong Zhang, Shi-Hai Xu

**Affiliations:** a Department of Chemistry, Jinan University, Guangzhou 510632, People’s Republic of China

## Abstract

In the title mononuclear complex, [Cu(C_14_H_10_O_3_)(C_12_H_8_N_2_)(H_2_O)], the Cu^II^ atom is five-coordinated by two N atoms from a 1,10-phenanthroline (phen) ligand, two O atoms from a benzilate ligand and one O atom from a water mol­ecule in a distorted square-pyramidal geometry. The crystal structure is stabilized *via* inter­molecular O—H⋯O and C—H⋯O hydrogen bonds, C—H⋯π inter­actions and π–π stacking inter­actions between the pyridine and benzene rings of neighboring phen ligands [centroid–centroid distances = 3.684 (2), 3.564 (2) and 3.380 (1) Å].

## Related literature

For related structures of benzilate compounds, see: Mora *et al.* (2003 ▶); Rojas *et al.* (2003 ▶).
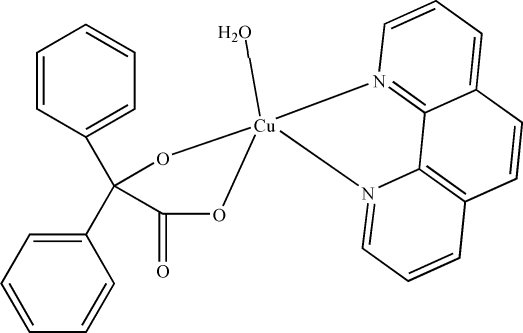


## Experimental

### 

#### Crystal data

[Cu(C_14_H_10_O_3_)(C_12_H_8_N_2_)(H_2_O)]
*M* *_r_* = 487.98
Triclinic, 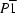

*a* = 7.4473 (15) Å
*b* = 9.757 (2) Å
*c* = 15.319 (3) Å
α = 102.99 (3)°
β = 98.39 (3)°
γ = 96.70 (3)°
*V* = 1060.1 (4) Å^3^
*Z* = 2
Mo *K*α radiation
μ = 1.07 mm^−1^
*T* = 293 K
0.30 × 0.26 × 0.21 mm

#### Data collection

Rigaku/MSC Mercury CCD diffractometer
Absorption correction: multi-scan (*REQAB*; Jacobson, 1998 ▶) *T*_min_ = 0.740, *T*_max_ = 0.807
8229 measured reflections
3802 independent reflections
2607 reflections with *I* > 2σ(*I*)
*R*_int_ = 0.049

#### Refinement

*R*[*F* ^2^ > 2σ(*F* ^2^)] = 0.073
*wR*(*F* ^2^) = 0.229
*S* = 1.09
3802 reflections
298 parameters
H-atom parameters constrained
Δρ_max_ = 0.73 e Å^−3^
Δρ_min_ = −1.54 e Å^−3^

### 

Data collection: *CrystalStructure* (Rigaku/MSC, 2002 ▶); cell refinement: *CrystalStructure*; data reduction: *CrystalStructure*; program(s) used to solve structure: *SHELXS97* (Sheldrick, 2008 ▶); program(s) used to refine structure: *SHELXL97* (Sheldrick, 2008 ▶); molecular graphics: *SHELXTL* (Sheldrick, 2008 ▶) and *DIAMOND* (Brandenburg, 1999 ▶); software used to prepare material for publication: *SHELXTL*.

## Supplementary Material

Crystal structure: contains datablocks I, global. DOI: 10.1107/S1600536809053483/hy2263sup1.cif

Structure factors: contains datablocks I. DOI: 10.1107/S1600536809053483/hy2263Isup2.hkl

Additional supplementary materials:  crystallographic information; 3D view; checkCIF report

## Figures and Tables

**Table 1:** Selected bond lengths (Å)

Cu1—O2	1.949 (4)
Cu1—O3	1.853 (4)
Cu1—N1	2.014 (4)
Cu1—N2	2.019 (5)
Cu1—O1*W*	2.476 (5)

**Table 2:** Hydrogen-bond geometry (Å, °)

*D*—H⋯*A*	*D*—H	H⋯*A*	*D*⋯*A*	*D*—H⋯*A*
O1*W*—H1*W*⋯O2^i^	0.82	2.07	2.883 (6)	171
O1*W*—H2*W*⋯O1^ii^	0.83	2.13	2.954 (4)	175
C17—H17⋯O1^iii^	0.93	2.41	3.312 (8)	162
C21—H21⋯*Cg*1^i^	0.93	2.46	3.267 (8)	146

Symmetry codes: (i) 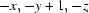; (ii) 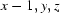; (iii) 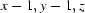. *Cg*1 is the centroid of the C9–C14 ring.

**Fig. 1.**
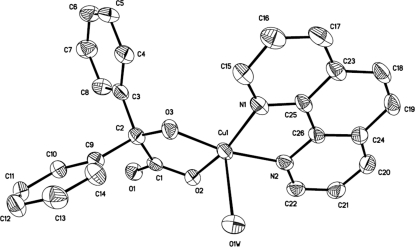
The molecular structure of the title compound, showing 50% probability displacement ellipsoids. H atoms have been omitted for clarity.

**Fig. 2.**
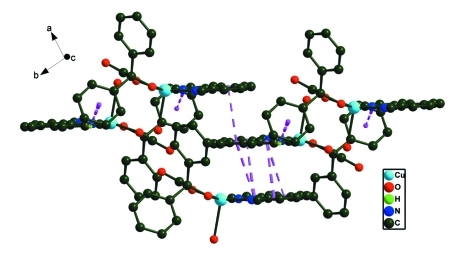
A packing view of the title compound. C—H···π interactions and π–π stacking interactions are shown as dashed lines.

## supplementary crystallographic information

### Comment

In the structural investigations of benzilate complexes, it has been found that the benzilic acid functions as a multidentate ligand with versatile binding and coordination modes (Mora *et al.*, 2003; Rojas *et al.*, 2003). In this paper, we report the structure of the title compound, a copper(II) complex obtained by the reaction of benzilic acid, 1,10-phenanthroline (phen) and copper chloride in an alkaline aqueous solution.

As depicted in Fig. 1, the Cu^II^ atom exists in a square-pyramidal environment, defined by two O atoms from one benzilate ligand, two N atoms from one phen ligand and one water molecule. The crystal structure is stabilized *via* intermolecular O—H···O and C—H···O hydrogen bonds, C—H···π interactions (Table 1) and π–π stacking interactions between the pyridine and benzene rings of neighboring phen ligands (Fig. 2), with the centroid–centroid distances of Cg2···Cg3^i^ = 3.684 (2), Cg3···Cg4^i^ = 3.564 (2) and Cg4···Cg4^ii^ = 3.380 (1) Å [Cg2, Cg3 and Cg4 are the centroids of the N1, C15, C16, C17, C23, C25 ring, the N2, C20, C21, C22, C24, C26 ring and the C18, C19, C23, C24, C25, C26 ring, respectively. Symmetry codes: (i) -x, -y, -z; (ii) -1-x, -y, -z].

### Experimental

A mixture of copper chloride (0.134 g, 1 mmol), benzilic acid (0.228 g, 1 mmol), phen (0.18 g, 1 mmol), NaOH (0.06 g, 1.5 mmol), EtOH (6 ml) and H_2_O (6 ml) was placed in a 23 ml Teflon-lined reactor, which was heated to 358 K for 8 h and then cooled to room temperature at a rate of 10 K h^-1^. The blue crystals obtained were washed with water and dried in air.

### Refinement

H atoms on C atoms were positioned geometrically and refined as riding atoms, with C—H = 0.93 Å and with *U*_iso_(H) = 1.2*U*_eq_(C). H atoms of water molecule were found in a difference Fourier map and refined as riding atoms, with *U*_iso_(H) = 1.5*U*_eq_(O). The highest residual electron density peak is located 0.73 Å from N2 and the deepest hole is located 1.54 Å from Cu1.

### Crystal data

**Table:**

[Cu(C_14_H_10_O_3_)(C_12_H_8_N_2_)(H_2_O)]	*Z* = 2
*M**_r_* = 487.98	*F*(000) = 502
Triclinic, *P*1	*D*_x_ = 1.529 Mg m^−^^3^
Hall symbol: -P 1	Mo *K*α radiation, λ = 0.71073 Å
*a* = 7.4473 (15) Å	Cell parameters from 2895 reflections
*b* = 9.757 (2) Å	θ = 2.4–27.9°
*c* = 15.319 (3) Å	µ = 1.07 mm^−^^1^
α = 102.99 (3)°	*T* = 293 K
β = 98.39 (3)°	Block, blue
γ = 96.70 (3)°	0.30 × 0.26 × 0.21 mm
*V* = 1060.1 (4) Å^3^	

### Data collection

**Table:**

Rigaku/MSC Mercury CCD diffractometer	3802 independent reflections
Radiation source: fine-focus sealed tube	2607 reflections with *I* > 2σ(*I*)
graphite	*R*_int_ = 0.049
ω scans	θ_max_ = 25.2°, θ_min_ = 3.1°
Absorption correction: multi-scan (REQAB; Jacobson, 1998)	*h* = −8→8
*T*_min_ = 0.740, *T*_max_ = 0.807	*k* = −11→11
8229 measured reflections	*l* = −18→18

### Refinement

**Table:**

Refinement on *F*^2^	Primary atom site location: structure-invariant direct methods
Least-squares matrix: full	Secondary atom site location: difference Fourier map
*R*[*F*^2^ > 2σ(*F*^2^)] = 0.073	Hydrogen site location: inferred from neighbouring sites
*wR*(*F*^2^) = 0.229	H-atom parameters constrained
*S* = 1.09	*w* = 1/[σ^2^(*F*_o_^2^) + (0.1409*P*)^2^] where *P* = (*F*_o_^2^ + 2*F*_c_^2^)/3
3802 reflections	(Δ/σ)_max_ < 0.001
298 parameters	Δρ_max_ = 0.73 e Å^−^^3^
0 restraints	Δρ_min_ = −1.54 e Å^−^^3^

### Fractional atomic coordinates and isotropic or equivalent isotropic displacement parameters (Å^2^)

**Table:**

	*x*	*y*	*z*	*U*_iso_*/*U*_eq_	
Cu1	−0.01419 (8)	0.32886 (6)	0.11584 (5)	0.0478 (3)	
O1	0.4514 (5)	0.5892 (4)	0.1612 (3)	0.0550 (10)	
O2	0.1851 (5)	0.4550 (4)	0.0914 (3)	0.0480 (9)	
O3	0.0740 (5)	0.4170 (4)	0.2375 (3)	0.0536 (10)	
N1	−0.1600 (6)	0.1655 (5)	0.1476 (4)	0.0507 (12)	
N2	−0.0958 (6)	0.2006 (5)	−0.0106 (3)	0.0471 (11)	
C1	0.3024 (7)	0.5192 (5)	0.1632 (4)	0.0430 (12)	
C2	0.2428 (7)	0.5060 (6)	0.2553 (4)	0.0458 (13)	
C3	0.3883 (8)	0.4467 (5)	0.3150 (4)	0.0505 (13)	
C4	0.3222 (9)	0.3619 (7)	0.3695 (5)	0.0617 (16)	
H4	0.1962	0.3390	0.3661	0.074*	
C5	0.4417 (11)	0.3116 (7)	0.4283 (5)	0.072 (2)	
H5	0.3951	0.2549	0.4637	0.087*	
C6	0.6278 (11)	0.3443 (7)	0.4350 (5)	0.0717 (19)	
H6	0.7076	0.3104	0.4748	0.086*	
C7	0.6948 (10)	0.4270 (8)	0.3826 (5)	0.0727 (19)	
H7	0.8211	0.4506	0.3874	0.087*	
C8	0.5765 (8)	0.4766 (7)	0.3222 (5)	0.0595 (16)	
H8	0.6249	0.5309	0.2860	0.071*	
C9	0.2267 (8)	0.6559 (6)	0.3114 (4)	0.0481 (13)	
C10	0.3750 (8)	0.7666 (6)	0.3423 (4)	0.0532 (14)	
H10	0.4902	0.7518	0.3289	0.064*	
C11	0.3524 (10)	0.8971 (7)	0.3922 (5)	0.0641 (17)	
H11	0.4519	0.9704	0.4108	0.077*	
C12	0.1844 (10)	0.9215 (7)	0.4153 (5)	0.0704 (18)	
H12	0.1710	1.0092	0.4509	0.085*	
C13	0.0366 (11)	0.8128 (8)	0.3845 (5)	0.079 (2)	
H13	−0.0778	0.8279	0.3990	0.095*	
C14	0.0569 (9)	0.6817 (7)	0.3324 (5)	0.0633 (17)	
H14	−0.0444	0.6103	0.3113	0.076*	
C15	−0.1857 (8)	0.1530 (7)	0.2300 (5)	0.0600 (16)	
H15	−0.1392	0.2287	0.2802	0.072*	
C16	−0.2811 (9)	0.0283 (8)	0.2425 (6)	0.0707 (19)	
H16	−0.2958	0.0222	0.3009	0.085*	
C17	−0.3527 (9)	−0.0840 (7)	0.1704 (6)	0.068 (2)	
H17	−0.4187	−0.1656	0.1788	0.081*	
C18	−0.3932 (8)	−0.1834 (6)	0.0027 (6)	0.0660 (19)	
H18	−0.4590	−0.2681	0.0068	0.079*	
C19	−0.3644 (8)	−0.1664 (6)	−0.0801 (6)	0.070 (2)	
H19	−0.4125	−0.2393	−0.1318	0.085*	
C20	−0.2216 (8)	−0.0133 (7)	−0.1706 (5)	0.0630 (17)	
H20	−0.2624	−0.0833	−0.2245	0.076*	
C21	−0.1223 (8)	0.1146 (7)	−0.1719 (5)	0.0626 (16)	
H21	−0.0952	0.1311	−0.2264	0.075*	
C22	−0.0627 (8)	0.2192 (6)	−0.0902 (4)	0.0540 (15)	
H22	0.0030	0.3054	−0.0918	0.065*	
C23	−0.3250 (7)	−0.0746 (6)	0.0826 (5)	0.0572 (17)	
C24	−0.2607 (8)	−0.0375 (6)	−0.0896 (5)	0.0545 (15)	
C25	−0.2280 (6)	0.0541 (5)	0.0764 (5)	0.0481 (14)	
C26	−0.1937 (7)	0.0717 (5)	−0.0108 (4)	0.0486 (14)	
O1W	−0.2525 (6)	0.4712 (4)	0.0741 (3)	0.0641 (12)	
H1W	−0.2344	0.5019	0.0299	0.096*	
H2W	−0.3373	0.5069	0.0958	0.096*	

### Atomic displacement parameters (Å^2^)

**Table:**

	*U*^11^	*U*^22^	*U*^33^	*U*^12^	*U*^13^	*U*^23^
Cu1	0.0511 (4)	0.0331 (4)	0.0568 (5)	0.0027 (3)	0.0079 (3)	0.0093 (3)
O1	0.051 (2)	0.052 (2)	0.062 (3)	−0.0036 (18)	0.013 (2)	0.018 (2)
O2	0.052 (2)	0.042 (2)	0.048 (2)	0.0046 (17)	0.0058 (18)	0.0105 (18)
O3	0.055 (2)	0.047 (2)	0.052 (3)	−0.0086 (18)	0.0080 (19)	0.0090 (19)
N1	0.048 (2)	0.036 (2)	0.069 (3)	0.006 (2)	0.014 (2)	0.012 (2)
N2	0.047 (2)	0.038 (2)	0.059 (3)	0.0138 (19)	0.009 (2)	0.014 (2)
C1	0.057 (3)	0.030 (3)	0.046 (3)	0.011 (2)	0.013 (3)	0.013 (2)
C2	0.044 (3)	0.038 (3)	0.055 (4)	0.003 (2)	0.011 (3)	0.012 (3)
C3	0.069 (3)	0.032 (3)	0.052 (3)	0.009 (2)	0.016 (3)	0.008 (2)
C4	0.070 (4)	0.050 (3)	0.066 (4)	0.001 (3)	0.012 (3)	0.020 (3)
C5	0.103 (5)	0.050 (4)	0.071 (5)	0.011 (4)	0.011 (4)	0.033 (4)
C6	0.092 (5)	0.060 (4)	0.070 (5)	0.033 (4)	0.005 (4)	0.023 (4)
C7	0.074 (4)	0.071 (5)	0.081 (5)	0.032 (4)	0.016 (4)	0.023 (4)
C8	0.059 (3)	0.058 (4)	0.069 (4)	0.017 (3)	0.015 (3)	0.024 (3)
C9	0.057 (3)	0.039 (3)	0.048 (3)	0.008 (2)	0.008 (3)	0.012 (3)
C10	0.058 (3)	0.042 (3)	0.055 (4)	0.000 (3)	0.002 (3)	0.011 (3)
C11	0.084 (4)	0.042 (3)	0.055 (4)	0.002 (3)	−0.005 (3)	0.002 (3)
C12	0.092 (5)	0.044 (4)	0.069 (5)	0.012 (4)	0.014 (4)	0.002 (3)
C13	0.094 (5)	0.076 (5)	0.080 (5)	0.042 (4)	0.036 (4)	0.019 (4)
C14	0.063 (4)	0.051 (4)	0.076 (5)	0.004 (3)	0.020 (3)	0.013 (3)
C15	0.061 (3)	0.046 (3)	0.074 (5)	0.003 (3)	0.017 (3)	0.014 (3)
C16	0.069 (4)	0.067 (4)	0.087 (5)	0.012 (3)	0.027 (4)	0.031 (4)
C17	0.055 (3)	0.049 (4)	0.110 (6)	0.009 (3)	0.022 (4)	0.037 (4)
C18	0.053 (3)	0.031 (3)	0.108 (6)	0.005 (3)	0.003 (4)	0.013 (4)
C19	0.054 (3)	0.033 (3)	0.107 (6)	0.012 (3)	−0.016 (4)	−0.004 (4)
C20	0.058 (3)	0.051 (4)	0.067 (5)	0.020 (3)	−0.008 (3)	−0.005 (3)
C21	0.063 (4)	0.059 (4)	0.064 (4)	0.024 (3)	0.000 (3)	0.012 (3)
C22	0.051 (3)	0.050 (3)	0.065 (4)	0.016 (3)	0.008 (3)	0.020 (3)
C23	0.038 (3)	0.038 (3)	0.097 (5)	0.011 (2)	0.011 (3)	0.018 (3)
C24	0.046 (3)	0.043 (3)	0.068 (4)	0.015 (2)	−0.005 (3)	0.006 (3)
C25	0.032 (2)	0.032 (3)	0.079 (4)	0.006 (2)	0.008 (3)	0.012 (3)
C26	0.043 (3)	0.032 (3)	0.068 (4)	0.014 (2)	0.000 (3)	0.008 (3)
O1W	0.067 (2)	0.062 (3)	0.072 (3)	0.021 (2)	0.019 (2)	0.026 (2)

### Geometric parameters (Å, °)

**Table:**

Cu1—O2	1.949 (4)	C11—C12	1.381 (10)
Cu1—O3	1.853 (4)	C11—H11	0.9300
Cu1—N1	2.014 (4)	C12—C13	1.382 (10)
Cu1—N2	2.019 (5)	C12—H12	0.9300
Cu1—O1W	2.476 (5)	C13—C14	1.385 (10)
O1—C1	1.241 (6)	C13—H13	0.9300
O2—C1	1.283 (7)	C14—H14	0.9300
O3—C2	1.396 (6)	C15—C16	1.402 (9)
N1—C15	1.333 (8)	C15—H15	0.9300
N1—C25	1.343 (8)	C16—C17	1.361 (10)
N2—C22	1.325 (8)	C16—H16	0.9300
N2—C26	1.377 (7)	C17—C23	1.411 (10)
C1—C2	1.567 (7)	C17—H17	0.9300
C2—C9	1.548 (8)	C18—C19	1.360 (10)
C2—C3	1.562 (8)	C18—C23	1.411 (10)
C3—C8	1.381 (8)	C18—H18	0.9300
C3—C4	1.400 (8)	C19—C24	1.445 (9)
C4—C5	1.382 (10)	C19—H19	0.9300
C4—H4	0.9300	C20—C24	1.379 (9)
C5—C6	1.369 (10)	C20—C21	1.380 (9)
C5—H5	0.9300	C20—H20	0.9300
C6—C7	1.363 (10)	C21—C22	1.402 (9)
C6—H6	0.9300	C21—H21	0.9300
C7—C8	1.389 (9)	C22—H22	0.9300
C7—H7	0.9300	C23—C25	1.403 (7)
C8—H8	0.9300	C24—C26	1.401 (9)
C9—C14	1.387 (8)	C25—C26	1.441 (9)
C9—C10	1.397 (8)	O1W—H1W	0.8214
C10—C11	1.373 (9)	O1W—H2W	0.8307
C10—H10	0.9300		
			
O3—Cu1—O2	85.53 (16)	C9—C10—H10	119.7
O3—Cu1—N1	91.89 (19)	C10—C11—C12	121.2 (6)
O2—Cu1—N1	163.11 (17)	C10—C11—H11	119.4
O3—Cu1—N2	169.49 (17)	C12—C11—H11	119.4
O2—Cu1—N2	98.71 (17)	C11—C12—C13	118.6 (6)
N1—Cu1—N2	81.2 (2)	C11—C12—H12	120.7
N1—Cu1—O1W	102.84 (17)	C13—C12—H12	120.7
N2—Cu1—O1W	86.73 (17)	C12—C13—C14	120.7 (7)
O2—Cu1—O1W	93.99 (16)	C12—C13—H13	119.7
O3—Cu1—O1W	102.66 (16)	C14—C13—H13	119.7
C1—O2—Cu1	113.1 (3)	C13—C14—C9	120.7 (6)
C2—O3—Cu1	115.2 (3)	C13—C14—H14	119.6
C15—N1—C25	117.9 (5)	C9—C14—H14	119.6
C15—N1—Cu1	127.8 (4)	N1—C15—C16	121.5 (7)
C25—N1—Cu1	114.2 (4)	N1—C15—H15	119.2
C22—N2—C26	116.9 (5)	C16—C15—H15	119.2
C22—N2—Cu1	130.3 (4)	C17—C16—C15	120.8 (7)
C26—N2—Cu1	112.7 (4)	C17—C16—H16	119.6
O1—C1—O2	123.3 (5)	C15—C16—H16	119.6
O1—C1—C2	121.6 (5)	C16—C17—C23	118.9 (6)
O2—C1—C2	115.1 (4)	C16—C17—H17	120.6
O3—C2—C9	110.0 (4)	C23—C17—H17	120.6
O3—C2—C3	109.6 (4)	C19—C18—C23	121.1 (6)
C9—C2—C3	106.6 (5)	C19—C18—H18	119.5
O3—C2—C1	109.5 (5)	C23—C18—H18	119.5
C9—C2—C1	109.1 (4)	C18—C19—C24	121.4 (6)
C3—C2—C1	112.0 (4)	C18—C19—H19	119.3
C8—C3—C4	117.2 (6)	C24—C19—H19	119.3
C8—C3—C2	125.7 (5)	C24—C20—C21	120.1 (6)
C4—C3—C2	117.0 (5)	C24—C20—H20	119.9
C5—C4—C3	120.9 (6)	C21—C20—H20	119.9
C5—C4—H4	119.5	C20—C21—C22	119.2 (6)
C3—C4—H4	119.5	C20—C21—H21	120.4
C6—C5—C4	120.7 (6)	C22—C21—H21	120.4
C6—C5—H5	119.6	N2—C22—C21	122.8 (6)
C4—C5—H5	119.6	N2—C22—H22	118.6
C7—C6—C5	119.2 (7)	C21—C22—H22	118.6
C7—C6—H6	120.4	C25—C23—C17	116.4 (6)
C5—C6—H6	120.4	C25—C23—C18	119.5 (7)
C6—C7—C8	120.7 (7)	C17—C23—C18	124.1 (6)
C6—C7—H7	119.6	C20—C24—C26	117.0 (6)
C8—C7—H7	119.6	C20—C24—C19	125.0 (6)
C3—C8—C7	121.2 (6)	C26—C24—C19	118.0 (6)
C3—C8—H8	119.4	N1—C25—C23	124.5 (6)
C7—C8—H8	119.4	N1—C25—C26	115.8 (5)
C14—C9—C10	118.2 (6)	C23—C25—C26	119.7 (6)
C14—C9—C2	118.6 (5)	N2—C26—C24	123.8 (6)
C10—C9—C2	123.2 (5)	N2—C26—C25	115.9 (5)
C11—C10—C9	120.6 (6)	C24—C26—C25	120.3 (5)
C11—C10—H10	119.7	H1W—O1W—H2W	109.4

### Hydrogen-bond geometry (Å, °)

**Table:**

Cg1 is the centroid of the C9–C14 ring.

**Table:**

*D*—H···*A*	*D*—H	H···*A*	*D*···*A*	*D*—H···*A*
O1W—H1W···O2^i^	0.82	2.07	2.883 (6)	171
O1W—H2W···O1^ii^	0.83	2.13	2.954 (4)	175
C17—H17···O1^iii^	0.93	2.41	3.312 (8)	162
C21—H21···Cg1^i^	0.93	2.46	3.267 (8)	146

Symmetry codes: (i) −*x*, −*y*+1, −*z*; (ii) *x*−1, *y*, *z*; (iii) *x*−1, *y*−1, *z*.
